# Effectiveness of Anthelmintic Treatments in Small Ruminants in Germany

**DOI:** 10.3390/ani12121501

**Published:** 2022-06-09

**Authors:** Katja Voigt, Maximilian Geiger, Miriam Carmen Jäger, Gabriela Knubben-Schweizer, Christina Strube, Yury Zablotski

**Affiliations:** 1 Clinic for Ruminants with Ambulatory and Herd Health Services, Centre for Clinical Veterinary Medicine, Ludwig-Maximilians-Universität (LMU) München, Sonnenstr. 16, 85764 Oberschleissheim, Germany; maxe.geiger@t-online.de (M.G.); gknubben@med.vetmed.uni-muenchen.de (G.K.-S.); y.zablotski@med.vetmed.uni-muenchen.de (Y.Z.); 2 Labor ParaDocs, Münchener Str. 101, 85737 Ismaning, Germany; m.jaeger@laborparadocs.de; 3 Institute for Parasitology, Centre for Infection Medicine, University of Veterinary Medicine Hannover, 30559 Hannover, Germany; christina.strube@tiho-hannover.de

**Keywords:** anthelmintic efficacy, faecal egg count reduction test, benzimidazoles, moxidectin, levamisole, monepantel, avermectins, closantel, sheep, goats

## Abstract

**Simple Summary:**

Parasitic worms are a major threat to the health and production of sheep and goats worldwide, particularly because many worms have become resistant to commonly applied drugs. This problem is well known around the world, but the situation is currently not well studied in Germany. This study was therefore performed to evaluate the effectiveness of treatments against infection with gastrointestinal nematodes. Faecal samples from animal groups were examined before and after treatment, the worm eggs in the feaces were counted, and the reduction in egg excretion following treatment was calculated. Eggs of *Haemonchus contortus* (barber’s pole worm) were stained to differentiate them from other gastrointestinal nematodes. Treatments were chosen and carried out by farmers together with their local veterinarian. Additional information was collected by questionnaires to check if the treatments were performed correctly. Reduced effectiveness was observed for all available drugs, but some showed better treatment success than others. The barber’s pole worm frequently survived treatments by most products. The high percentage of treatment failures is highly concerning, and it is necessary to develop and/or apply alternative methods of worm control to prevent the deterioration of this situation.

**Abstract:**

Widespread anthelmintic resistance is a concern for small ruminant health and production worldwide. The current situation regarding anthelmintic efficacy is, however, not very well studied in Germany. Thus, a nationwide field study was undertaken to assess the effectiveness of 253 treatments performed in 223 small ruminant flocks by faecal egg count reduction test (FECRT) using pooled samples and a modified McMaster method. The percentage of *Haemonchus contortus* and non-*Haemonchus* eggs was determined by fluorescence microscopy following peanut agglutinin–fluorescein isothiocyanate staining. Treatments were chosen and performed by farmers together with their local veterinarian, and potentially confounding factors for FECRT results were addressed as far as possible by rigorous inclusion criteria. Reduced effectiveness was observed for treatments with all examined anthelmintic classes, but treatments with benzimidazoles and moxidectin showed significantly poorer results than monepantel, a closantel and mebendazole combination, and levamisole. Low case numbers precluded reliable assessment of avermectins. Unsuccessful treatments were frequently associated with the survival of *H. contortus*, but this was also observed for non-*Haemonchus* genera. The results are highly concerning, and sustainable approaches to parasite control are urgently needed to prevent further deterioration of this situation.

## 1. Introduction

Anthelmintic resistance in small ruminant gastrointestinal nematodes (GIN) has reached alarming levels worldwide, with a particularly concerning situation in the southern hemisphere [1,2,3]. In comparison, the situation in Europe has been less severe, albeit serious, for many years. It is, however, evolving rapidly, and the resistance reported from European countries now involves all anthelmintic classes and all common GIN genera [4,5]. A recent meta-analysis of 197 publications from 22 European countries reported widespread resistance identified in Europe in the last decade, particularly for benzimidazoles, but also for levamisole and macrocyclic lactones, with moxidectin affected to a lesser extent. In contrast, the frequency of resistance against monepantel, although present, was still considered low. There were insufficient data to draw conclusions on the resistance of GIN in Europe against closantel [5].

The extent of anthelmintic resistance in small ruminant GIN in Germany is currently not very well studied. Resistance is, however, well known for benzimidazoles [6,7,8,9,10,11,12] and has also been reported for avermectins [9,10], moxidectin [8,9] and levamisole [10,11], including multiple resistance on some farms [9,10,11], and involving all major GIN species [6,9,10,11,12]. In neighbouring Austria, the frequency of benzimidazole resistance alleles has recently been shown to be extremely high, particularly for *Haemonchus contortus* and *Trichostrongylus colubriformis* [13]. To date, there are no published reports studying the efficacy of monepantel or closantel in German small ruminant flocks. At the time of writing, licensed anthelmintic classes for treatment of GIN infections in sheep in Germany include macrocyclic lactones (milbemycins: moxidectin; avermectins: ivermectin, doramectin, eprinomectin), benzimidazoles (albendazole, fenbendazole, oxfendazole), imidazothiazoles (levamisole), amino-acetonitrile derivatives (monepantel) plus a combination product containing a narrow-spectrum salicylanilide (closantel) and a benzimidazole component (mebendazole). Until 2021, when eprinomectin was licensed for use in goats in Germany, all treatments of this species had to be carried out under cascade regulations [14]. Due to different pharmacokinetics of anthelmintic drugs in goats in comparison to sheep or cattle, higher dose rates are generally required to reach therapeutic levels in this species [14,15,16,17,18,19,20,21]. An additional anthelmintic class, spiroindoles, with its active compound derquantel, is available in combination with the macrocyclic lactone abamectin in a number of countries including the UK in Europe [22,23], but is not licensed in Germany.

Resistance to one substance within an anthelmintic class generally leads to side resistance [24,25], i.e., resistance to all other drugs within this class, although various drugs or sub-groups within one class can have different potency. This is particularly the case for macrocyclic lactones, with a higher potency of the milbemycins such as moxidectin in comparison to avermectins [26]. While there is some level of cross-resistance between moxidectin and avermectins [27,28], these two groups should be assessed separately when studying anthelmintic resistance to account for differences in their pharmacokinetics, potency, toxicity, and probable resistance mechanisms [28].

Methods to diagnose anthelmintic resistance have recently been reviewed by Gilleard et al. (2021) [3]. Despite major scientific advances in this field since the publication of the World Association for the Advancement of Veterinary Parasitology (WAAVP) guidelines in 1992, which defined standardized methods for the detection of anthelmintic resistance in ruminants [29], and their re-evaluation in 2006 [30], the faecal egg count reduction test (FECRT) and its recent adaptations remain the only method applicable to all anthelmintic classes and suitable for field conditions [26]. New WAAVP guidelines for the diagnosis of anthelmintic resistance in ruminants are currently being developed, but have not yet been published at the time of writing [31,32]. According to Charlier et al. (2022) [31], these are likely to include guidelines for the performance of a FECRT in farmed ruminants consistent with the latest recommendations by Kaplan (2020) [26]. General guidelines for the evaluation of efficacy of anthelmintics have recently been published [32]. While these mainly focus on trial design to assess the efficacy of new anthelmintic compounds, these recommendations can also aid the design of future studies assessing the efficacy of existing anthelmintics. According to previous WAAVP guidelines, calculation of faecal egg count reduction (FECR) is based on pre- and post-treatment egg counts of 15 individual animals in comparison to a control group of the same size. Reduced efficacy is then defined as FECR < 95% with the lower limit of the 95% confidence interval < 90% [29,33]. Geurden et al. (2022) recommend that evaluation of anthelmintic efficacy in field studies should be based primarily on FECR between pre- and post-treatment samples from treated animals only. These authors recommend a minimum efficacy of 90% FECR for new anthelmintic compounds [32]. A threshold of 95% FECR as the definition for an effective treatment is, however, set in the latest recommendations regarding the evaluation of potential anthelmintic resistance in farmed ruminants [26]. In combination with traditional [34,35] or molecular methods [36,37] for genus or species differentiation, the FECRT can also provide very useful information regarding genus- or species-specific resistance in small ruminant GIN. Several studies have recently evaluated the use of pooled faecal samples in comparison to the traditional FECRT based on individual egg counts and have concluded that pooling is a suitable approach to achieve cost-effective and yet viable results to assess anthelmintic efficacy under field conditions [26,38,39]. Correlation of mean values obtained from individually examined samples and the respective pooled FEC results was very high, particularly when a pool size of five animals per pool was used [38]. For the use of pooled samples, however, the sensitivity of diagnostic methods and baseline egg counts need to be carefully considered, as low individual egg excretion may result in erroneous zero egg counts when examining pooled samples with low baseline egg counts [29]. Calculation of 95% confidence intervals is not possible for a pooled FECRT approach, and results thus need to be interpreted accordingly [26]. Irrespective of individual or composite faecal egg count approaches, confounding factors (factors other than anthelmintic resistance) always need to be taken into account when interpreting FECR [40]. Non-standardized approaches are therefore ultimately a measure for the effectiveness of performed treatments rather than a direct measurement of anthelmintic resistance, but they allow for conclusions regarding anthelmintic efficacy if potential confounding factors are adequately controlled [26,40].

Anecdotal evidence in Germany suggests that the current effectiveness of anthelmintic treatments in small ruminants is frequently compromised, and reduced effectiveness is suspected for all available anthelmintic classes. However, no recent studies have so far been undertaken to assess this situation. This large-scale, nationwide study was thus designed to screen a large number of farms to evaluate the effectiveness of anthelmintic treatments performed in the field in German small ruminant flocks, using a composite sampling approach for the assessment of pre- and post-treatment egg counts as well as potential changes in the pre- and post-treatment percentage of *H. contortus* and other strongyle eggs to gain additional information on the potential selective survival of *H. contortus* or other genera.

## 2. Materials and Methods

Diagnostic faecal samples were submitted voluntarily by farmers or veterinarians between September 2019 and December 2020 following nationwide advertisement of the study via veterinary and agricultural organisations, breeding associations, and agricultural and veterinary journals. To encourage participation, all diagnostic tests were carried out free of charge. Participants expressed their interest by contacting the second author (M.G.) and subsequently received a sampling kit by mail. This consisted of three sample containers, a pair of gloves, an instruction sheet, a questionnaire and consent form, and a pre-paid, pre-addressed return label for submission to the laboratory of the Clinic for Ruminants, LMU Munich, Germany by mail. Inclusion criteria for participation were farm location in Germany, a minimum flock size of 15 sheep or goats of all age groups, and access to pasture.

### 2.1. Sample Collection and Coproscopical Methods

Participants were asked to collect three pooled faecal samples from five individual animals per pool, with sample collection from the rectum or immediately following de-faecation, and to fill a 120 mL screw-top container tightly packed to the brim, collecting similar amounts of faeces from each animal. Following receipt of the samples at the laboratory, they were either examined on the same day or refrigerated at 4 °C until processing. The mean time between on-farm sampling and laboratory processing was 6.2 days (SD: 2.8; median: 6; min: 0; max: 18). Each pool was examined individually using a modified McMaster technique to determine the number of strongyle eggs per gram of faeces (epg). Following thorough homogenization of the pooled sample using a spatula, 4 g faeces were weighed and suspended in approximately 20 mL saturated sodium chloride solution (specific gravity: 1.2), transferred through a sieve into a measuring cylinder, and the sieve was flushed with additional saturated sodium chloride solution to reach a final volume of 60 mL. This suspension was mixed thoroughly and immediately transferred to fill three McMaster counting grids. These were left to stand for a minimum of three minutes. All three counting grids were examined and strongyle eggs counted for each sample at 100× magnification. One egg counted thus equalled 33.3 epg [41]. Egg counts were subsequently rounded to whole numbers.

In cases where each pool represented a distinct age or management group, these were evaluated individually. If two or all three pools had been taken from the same management group, faecal egg counts were determined individually for each pool, and the arithmetic mean of the pools’ egg counts was calculated for this animal group. Between five and 15 animals thus contributed to the results of each management group. Any groups with results exceeding 200 epg were eligible for follow-up examination should the participant wish to perform anthelmintic treatments. Samples > 200 epg were also subjected to fluorescence microscopy following staining with peanut agglutinin conjugated with fluorescein isothiocyanate (PNA-FITC; Sigma Aldrich, St. Louis, MO, USA) [35]. The suspension previously prepared for the McMaster examination was thoroughly mixed and immediately transferred to fill a 12 mL centrifuge vial with 13 mL suspension to the brim, which was then covered with a cover slip and centrifuged at 1000× *g* for five minutes. All material adhering to the cover slip was flushed into a 2 mL microcentrifuge tube using a pipette with 2 mL tap water and centrifuged at 1300× *g* for three minutes. The supernatant was discarded, and the sediment was mixed with 100 µL 0.5% PNA-FITC solution prepared from 1 mg PNA-FITC in 199 mL phosphate-buffered saline. The sample was then kept under careful mechanical agitation at room temperature for one hour, subsequently left to stand for another hour, and then centrifuged at 1300× *g* for two minutes. The supernatant was discarded and 10 µL of the sediment were transferred onto a glass slide. A small drop of fluorescent mounting media (Merck KGaA, Darmstadt, Germany) was added to the sample, which was then covered with a cover slip and examined at 495 nm and at 200× magnification. Depending on the number of strongyle eggs present in the sample, a minimum of ten and a maximum of 100 eggs were examined for bright green fluorescence and counted. Strongyle eggs with fluorescence were identified as *H. contortus*, eggs without fluorescence were classified as non-*Haemonchus* (other) genera, and the percentage of *H. contortus* eggs in the sample was subsequently calculated [35].

### 2.2. Anthelmintic Treatments and Post-Treatment Samples

All treatment decisions were taken, anthelmintic products chosen, and treatments performed by the participants following the advice of their local veterinarian, since anthelmintics are prescription-only drugs in Germany. Farmers were asked to collect corresponding post-treatment samples 10–14 days following anthelmintic treatment [29] if they opted to treat their animals. All follow-up samples were subjected to the modified McMaster method, and PNA-FITC fluorescence microscopy was subsequently performed on all follow-up samples positive for strongyle eggs.

### 2.3. Questionnaires

Questionnaires were completed by each participant upon sample submission, specifying farm location, species, breed, flock size, farm type, pasture conditions, and sample identification/animal group(s) sampled. Alongside the submission of post-treatment samples, additional information was collected regarding anthelmintic treatment, including the type of product used, the application route, the dose rate, and the number of days between treatment and post-treatment sample collection. Participants were also asked to specify any potential observations or suspicions of prior ineffective treatments on the farm, alongside the name of the suspectedly ineffective anthelmintic product(s). Based on the animal weights and doses stated in the questionnaire, treatments were classified as “correct”, “underdosed”, or “overdosed”. For sheep, a correct dose was defined as by product data sheet instructions. Since no licensed anthelmintics were available for goats in Germany at the time of the study, goats were treated under cascade regulations using licensed products for sheep or cattle. For the use of levamisole in goats, a correct dose rate was defined as 1.5× the licensed sheep dose, while 2× the licensed sheep dose was defined as correct for goats for all other anthelmintic classes [14]. Any dose rates lower than these were classified as “underdosed”, while doses exceeding the above definitions by more than 50% were classified as “overdosed”. Anthelmintic compounds which were the sole representative of an anthelmintic class are subsequently referred to by active ingredient for better readability. Different substances belonging to the same anthelmintic class were categorized and are subsequently referred to by anthelmintic class because of their similarities in mode of action and resistance mechanisms [26].

### 2.4. Assessment of Treatment Effectiveness

Inclusion criteria for assessment of treatment effectiveness were: >200 epg in initial submission, use of an anthelmintic product licensed for treatment of GIN, correct dose rate (or higher) for the relevant small ruminant species, and corresponding post-treatment sample collection from identical animal groups as for the initial submission. Collection of these samples between seven and 21 days post treatment was considered acceptable depending on the anthelmintic class used [31]. For the purposes of this study, the accepted treatment-to-sampling interval was set at 7–16 days for levamisole, 10–21 days for avermectins and moxidectin, 10–19 days for monepantel and benzimidazoles, and 8–19 days for the closantel and mebendazole combination product.

Reduction between the pre- and post-treatment egg counts was calculated based on mean egg counts in the pre- and post-treatment sample submissions and rounded to whole numbers. In cases where post-treatment egg counts exceeded pre-treatment results, reduction was defined as 0%. Due to the use of pooled samples, it was not possible to calculate confidence intervals (CI). To assess the effectiveness of the performed treatments we used a cautious interpretation based on the recommendations by Kaplan (2020) for FECRT without calculation of CI [26] but defined effectiveness as ≥95% rather than >95%. The following interpretation was thus used to assess the level of (in)effectiveness of performed treatments, and the associated potential presence of suspected reduced drug efficacy following consideration of potentially confounding factors:

- FECR ≥ 95%: treatment effective, no evidence of resistance
- FECR ≥ 90% and < 95%: reduced efficacy, suspected resistance
- FECR ≥ 80% and < 90%: reduced efficacy, resistance is likely
- FECR < 80%: ineffective, resistance is highly likely

### 2.5. Statistical Analyses

Data analysis was performed using the R version 4.0.3 (Vienna, Austria) [42,43]. In cases where farmers treated more than one management group with the same anthelmintic compound, the arithmetic mean of FEC and *H. contortus* percentages of these groups were used for flock-level analyses regarding treatment success for the individual anthelmintic categories. In cases where farmers used different anthelmintic classes in different animal groups, each treatment was evaluated separately for the respective anthelmintic category. The number of evaluated treatments thus exceeds the number of participating flocks.

The data were checked for normality using the Shapiro–Wilk test, and non-parametric tests were subsequently used due to non-normally distributed data. For the initial, pre-treatment samples, a negative binomial counts model was used to assess potential differences between the two small ruminant species regarding egg excretion. Potential differences between sheep and goats regarding the percentage of *H. contortus* eggs were examined by quasibinomial generalized linear regression. Simple logistic regressions were performed to test for potential associations of small ruminant species or previously suspected unsuccessful treatments (as by questionnaire results) with treatment success (FECR ≥ 95%). Potential differences regarding FECR between the anthelmintic categories were assessed by Kruskal–Wallis test followed by post-hoc pairwise comparisons using Dunn test with Holm-correction for multiple comparisons. A quasibinomial generalized linear regression was applied to predict probabilities of FECR for the various anthelmintic classes. Pre- and post-treatment percentages of *H. contortus* and non-*Haemonchus* strongyle eggs were examined for each anthelmintic category using paired Wilcoxon tests. In all analyses, *p*-values ≤ 0.05 were considered significant, while *p*-values > 0.05 and < 0.1 were considered a tendency.

## 3. Results

### 3.1. Sample and Flock Characteristics

Initial sample sets were screened from 398 sheep flocks, 101 goat herds, and three mixed flocks from 15 of the 16 German federal states. Of these, a sub-set of 197 sheep flocks, 40 goat herds, and 3 mixed flocks from 14 federal states (except the city states of Berlin and Bremen) took part in the follow-up examination after anthelmintic treatment of their animals. Since only animal groups with an initial result > 200 epg were eligible for this second examination, the number of submitted follow-up samples and examined animal groups varied between one and three per flock. Participants of the follow-up examination submitted 412 ovine pooled samples from 349 distinct ovine management groups, and 95 caprine pooled samples from 93 caprine management groups.

The size of participating sheep flocks ranged from 5 to 1900 adult ewes (mean: 184, median: 54), while between 5 and 300 adult females were kept in the participating goat herds (mean: 40, median: 19). In the majority of cases, adult animals were sampled (sheep: 211/349 animal groups, 60.5%; goats: 64/93, 68.8%), while 30.4% (106/349) of the examined ovine and 12.9% (12/93) of the caprine groups were under 12 months of age. The remaining 32 ovine (32/349, 9.2%) and 17 caprine groups (17/93, 18.3%) were of mixed age.

In around two thirds of the flocks the farmers did not suspect any insufficient effectiveness of previously performed anthelmintic treatments (sheep: 130/197 flocks, 66.0%; goats: 25/40 herds, 62.5%; mixed flocks: 2/3, 66.7%). In the remaining flocks, farmers’ suspicions included all anthelmintic classes licensed in Germany, with some farmers naming more than one anthelmintic class, and moxidectin and benzimidazoles being the most frequently mentioned products for both species.

### 3.2. Compliance with Inclusion Criteria, Anthelmintic Treatments, and Pre-Treatment Coproscopical Results

Evaluation of the correct dose rate relied on the participants’ declarations in the questionnaire. According to this information, 341 of the 349 ovine groups (97.7%) received a correct dose of anthelmintic, while 8 groups (2.3%) from 5 (2.5%) of the 197 flocks were overdosed. Overdosed treatments were subsequently retained in the study. In contrast, 17 of the 93 caprine groups (18.3%) from ten of the 40 herds (25%) were treated using only the licensed sheep dose (8 herds) or insufficiently increased dose rates for goats (2 herds) and were thus classified as underdosed. These ten treatments were subsequently excluded from further analyses. Treatments using only the licensed sheep dose without any increase included 3× moxidectin, 2× monepantel, 2× levamisole, and 1× ivermectin, while moxidectin was used at 1.5× the ovine dose in one herd, and monepantel was applied at 1.3× the ovine dose in another. Three of these treatments (1× moxidectin, 1× levamisole, and 1× ivermectin) were classified as unsuccessful, while the other seven reached FECR values ≥ 95% despite being considered underdosed.

Following exclusion of a further four caprine and ten ovine management groups for farmers not complying with the accepted treatment-to-sampling interval for the applied anthelmintic compounds, the effectiveness of the applied treatments could be evaluated based on the results of 339 ovine and 72 caprine animal groups originating from 223 flocks (192 sheep flocks, 29 goat herds and 2 mixed flocks, of which, however, only one species was examined [1× sheep, 1× goats]. These treatments are thus subsequently referred to by species).

Some farmers chose to use different anthelmintics in their respective animal groups, while others used the same product in two or three of the examined groups. Hence, the number of anthelmintic classes used per flock varied between one and three. On the flock level, the 223 participants thus conducted 253 treatments (sheep: 218; goats: 35). Evaluation of these treatments was based on 15 animals (3 pooled samples) in 75 cases (29.6%), on 10 animals (2 pools) in 71 cases (28.1%) and on 5 animals (1 pool) in 107 cases (42.3%). Table 1 gives an overview of the use of the various anthelmintic classes by the participants. Oral preparations were used for the vast majority of treatments with the exception of six ovine and three caprine cases. Injectable avermectins were applied in five sheep flocks and two goat herds, while an avermectin product for pour-on application was used in one goat herd, and injectable levamisole was used in one sheep flock. Overall, moxidectin was the most frequently used anthelmintic compound in both species (sheep: 39.4% [86/218], goats: 40% [14/35] of the performed treatments).

Detailed results of the individual treatments are presented in Supplementary Materials. The pre-treatment faecal egg count (based on the arithmetic mean of the examined pools) for the 218 ovine treatments ranged from 233 epg to 21,933 epg, with a mean egg count of 1314 and a median of 726. For goats (*n* = 35 treatments), the initial egg count ranged from 233 epg to 6078 epg (mean: 1406; median: 950). Pre-treatment percentage of *H. contortus* eggs (based on the arithmetic mean of the examined pools) ranged from 0 to 100% in both species. Less than ten eggs were recovered for fluorescence microscopy for pre-treatment evaluation from six ovine flocks, their results are therefore not included. For the remaining 212 ovine treatments, the mean pre-treatment percentage of *H. contortus* eggs was 38.9% (median: 29.5%). No *H. contortus* eggs were detected in 32 ovine cases (32/212, 15.0%), while 100% *H. contortus* were identified in five (5/212, 2.4%). For goats, the mean pre-treatment *H. contortus* percentage was 52.5% (median: 54.0%) with no *H. contortus* eggs detected in 3 cases (3/35, 8.6%) and 100% of strongyle eggs identified as *H. contortus* in one (1/35, 2.9%). There was no statistically significant difference between the two small ruminant species regarding the initial egg counts (*p* = 0.681), but sheep samples contained a lower percentage of *H. contortus* eggs prior to treatment (*p* = 0.034; odds ratio (OR): 0.74; 95% CI: 0.57–0.99).

### 3.3. Post-Treatment Coproscopical Results and Assessment of Treatment Effectiveness

Post-treatment faecal egg counts ranged from < 33.3 epg in both species to 7966 in sheep (mean: 252; median: 14) and to 2900 in goats (mean: 252; median: 33). No *H. contortus* eggs were detected following treatment in 137 (62.8%) of the 218 ovine and in 20 (57.1%) of the 35 caprine cases (mean [sheep]: 28.9%; mean [goats]: 25.7%; median: [both species]: 0.0%). *H. contortus* eggs accounted for 100% in post-treatment samples in 15.1% (33/218) of the ovine and in 14.3% (5/35) of caprine cases.

The performed treatments were successful as defined by FECR ≥ 95% in 142 of the 218 cases (65.1%) for sheep and in 19 of the 35 cases (54.3%) for goats, with no significant difference between the two small ruminant species (*p* = 0.218).

No statistically significant association was shown for observed or suspected prior treatment failure(s) stated in the questionnaire and the actual treatment success in this study (sheep: *p* = 0.125; goats: *p* = 0.748). However, the majority of farmers who reported suspected previous treatment failure(s) (*n* = 72 flocks naming a total of 92 suspected failures) used different anthelmintic classes from the suspectedly ineffective product(s) for this study. It was thus not possible to statistically assess the validity of these farmer perceptions for the individual anthelmintic categories due to low case numbers. Of the twenty-four flocks treated with one (*n* = 21) or two (*n* = 3) suspectedly previously ineffective product(s), eight (33.3%) were diagnosed with treatment failure(s) for one (*n* = 7) or both (*n* = 1) of the applied anthelmintic class(es). Table 2 describes the number of flocks with suspected previous ineffective treatments by anthelmintic category, the number of farmers using the suspectedly ineffective anthelmintic class in this study, and the number of unsuccessful treatments for each category.

Across both small ruminant species and on the flock level, 92 of the 253 performed treatments (36.4%) did not achieve a FECR ≥ 95%, and these treatment failures included all anthelmintic classes used by the participants, albeit to a different degree. Table 3 summarizes the observed proportion and percentage of unsuccessful treatments for both small ruminant species as defined by FECR < 95% for the various anthelmintic classes chosen by the participants.

The observed FECR achieved by the performed treatments was variable and ranged from 100% to 0%. For unsuccessful treatments with moxidectin (sheep: *n* = 39; goats: *n* = 3), the mean FECR was 60.7% (median: 73%; SD 30.2; range: 0–93%) for sheep and 76.3% (median: 71%; SD: 12.0%; range: 65–93%) for goats; for avermectins (sheep: *n* = 3; goats: *n* = 3) 27.7% for sheep (median: 0%; SD: 39.1; range: 0–83%) and 90.9% for goats (median: 89%; SD: 1.4; range: 89–90%); for levamisole (sheep: *n* = 6; goats: *n* = 3) 89.7% in sheep (median: 91%; SD: 3.0; range: 83–93%) and 87.3% in goats (median: 88%, SD: 0.9; range: 86–88%); for monepantel (sheep: *n* = 3; goats: *n* = 1) 81.7% in sheep (median: 85%; SD: 10.9; range: 67–93%) and 72.0% in goats; for benzimidazoles (sheep: *n* = 23; goats: *n* = 6) 37.9% for sheep (median: 43%; SD: 34.7; range: 0–93%) and 56.8% in goats (median: 70%; SD: 35.7; range: 0–93%). The closantel and mebendazole combination was only applied in sheep and treatments were classified as unsuccessful in two cases, with a mean FECR of 53.5% (range: 50–57%). Table 4 shows descriptive statistics of the observed FECR for all examined flocks in the various anthelmintic categories, including successful und unsuccessful treatments, and for both small ruminant species.

**Figure 1:**
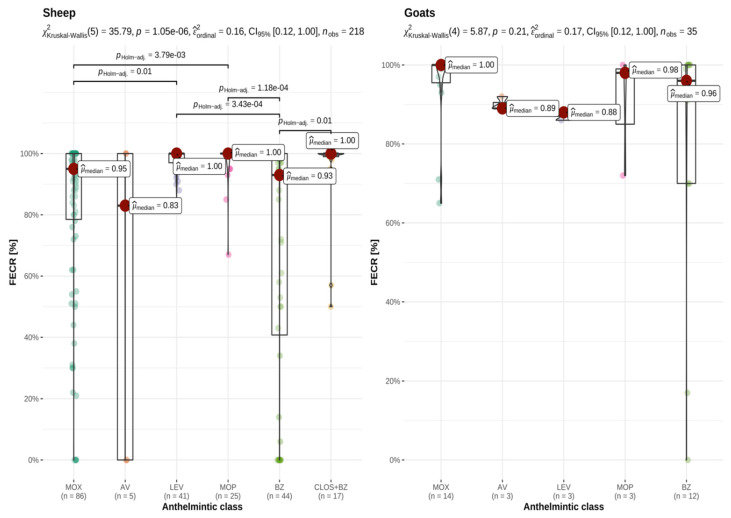
Faecal egg count reduction (FECR) following 253 anthelmintic treatments (sheep: 218; goats: 35) performed in 223 small ruminant flocks (sheep: 193; goats: 30), and paired comparison between anthelmintic categories. *n* = number of flocks using anthelmintic category; MOX = moxidectin; AV = avermectins, LEV = levamisole; MOP = monepantel; BZ = benzimidazoles; CLOS + BZ = closantel and mebendazole combination product; X^2^_Kruskal-Wallis_(5) = Kruskal–Wallis chi-squared statistics with the degrees of freedom, ε^2^_ordinal_ = effect size with CI_95%_ = 95% confidence intervals for the effect size; *n*_obs_ = number of observations. Interpretation of ε^2^_ordinal_ = 0.16: large effect size [44]. Explanation of scientific notation for *p*-values: 1.05e-06 is equivalent to *p* < 0.001.

There were significant differences in the effectiveness of treatments carried out using the different anthelmintic classes in sheep (*p* < 0.001), but no statistical significance could be shown for goats due to low case numbers (*p* = 0.21). Figure 1 illustrates the FECR for these treatments in the participating sheep flocks and goat herds by anthelmintic class, and pairwise comparisons between the various anthelmintic categories. In sheep, monepantel, levamisole, and the closantel and mebendazole combination showed significantly superior results in comparison to benzimidazoles and, in part, moxidectin. Avermectins showed the lowest median FECR, but these compounds were only used in five sheep flocks; low case numbers therefore precluded reliable statistical assessment of this anthelmintic group. Low case numbers also did not allow for reliable statistical assessment of potential differences in faecal egg count reduction following treatments with the different anthelmintic classes in goats.

**Figure 2:**
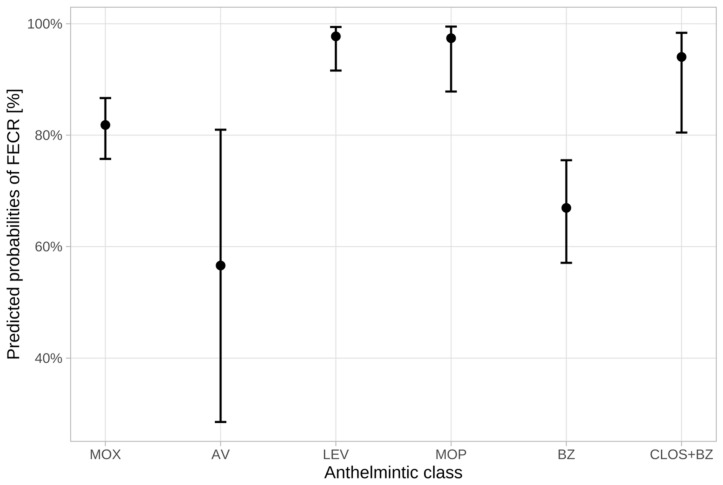
Predicted probabilities of faecal egg count reduction (FECR) in sheep for the various anthelmintic categories, with error bars indicating the 95% confidence interval. MOX = moxidectin; AV = avermectins, LEV = levamisole; MOP = monepantel; BZ = benzimidazoles; CLOS + BZ = closantel and mebendazole.

Since low case numbers resulted in wide 95% confidence intervals for the assessment of the various treatments in goats, no statistically significant differences could be shown for the various treatments in this species. Predicted probabilities of FECR for treatments with the different anthelmintic classes and results of pairwise comparisons of these predicted probabilities for the anthelmintic categories are therefore only presented for sheep. These results are illustrated and summarized in Figure 2 and Table 5. Pairwise comparisons showed significantly superior predicted probability of FECR for levamisole, monepantel, and the closantel and mebendazole combination in comparison to benzimidazoles, avermectins and, in part, moxidectin. Moxidectin was only significantly superior to benzimidazoles.

### 3.4. Assessment of Post-Treatment Survival of Haemonchus Contortus and Other GIN

**Figure 3:**
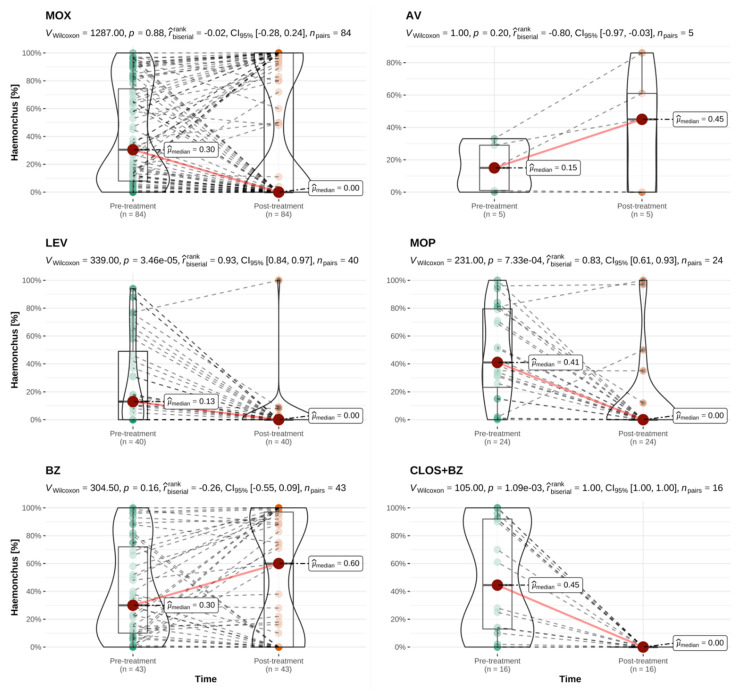
Paired comparison of the pre- and post-treatment percentage of *Haemonchus contortus* eggs for treatments performed in sheep, by anthelmintic category. This analysis included all ovine treatments irrespective of their classification as successful or unsuccessful. MOX = moxidectin; AV = avermectins, LEV = levamisole; MOP = monepantel; BZ = benzimidazoles; CLOS + BZ = closantel and mebendazole; *n* = number of flocks treated per category with available paired results. r^rank^_biserial_ = effect size with CI_95%_ = 95% confidence interval; *n*_pairs_ = number of paired results. Interpretation of r^rank^_biserial_: < 0.05: tiny; ≥0.05 and <0.1: very small; ≥0.1 and <0.2: small; ≥0.2 and <0.3: medium; ≥0.3 and <0.4: large; ≥0.4: very large [45]. Explanation of scientific notation for *p*-values: 3.46e-05 and 7.33e-04 are both equivalent to *p* < 0.001; 1.09e-03 is equivalent to *p* = 0.001.

**Figure 4:**
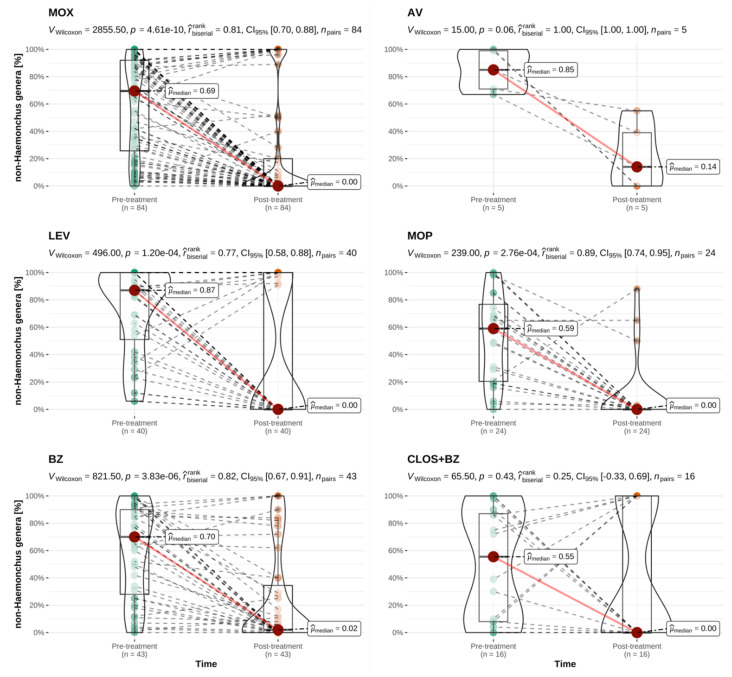
Paired comparison of **the** pre- and post-treatment percentage of non-*Haemonchus* strongyle eggs for treatments performed in sheep, by anthelmintic category. This analysis included all ovine treatments irrespective of their classification as successful or unsuccessful. MOX = moxidectin; AV = avermectins, LEV = levamisole; MOP = monepantel; BZ = benzimidazoles; CLOS + BZ = closantel and mebendazole; *n* = number of flocks treated per category with available paired results. r^rank^_biserial_ = effect size with CI_95%_ = 95% confidence interval; *n*_pairs_ = number of paired results. Interpretation of r^rank^_biserial_: <0.05: tiny; ≥0.05 and <0.1: very small; ≥0.1 and <0.2: small; ≥0.2 and <0.3: medium; ≥0.3 and <0.4: large; ≥0.4: very large [45]. Explanation of scientific notation for *p*-values: 4.61e-10, 1.20e-04, 2.76e-04 and 3.83e-06 are all equivalent to *p* < 0.001.

Comparisons of the pre- and post-treatment percentage of *H. contortus* and non-*Haemonchus* strongyle eggs were performed for each anthelmintic category to include all treatments irrespective of their classification as successful or unsuccessful. The only anthelmintic product to reduce the *H. contortus* percentage to zero in all treated sheep flocks was the combination of closantel and mebendazole. In all cases where strongyle eggs were present in post-treatment samples following the use of this product, these were identified as non-*Haemonchus* genera. In sheep, a significant reduction in *H. contortus* percentage across all examined flocks was only achieved following treatments with levamisole, monepantel and the closantel and mebendazole combination (Figure 3). A significant reduction in the percentage of other, non-*Haemonchus* eggs was seen for treatments with all anthelmintic classes except for closantel and mebendazole. However, survival of non-*Haemonchus* genera was also observed following a number of treatments with the other anthelmintic categories (Figure 4). Selective survival of *H. contortus* was thus frequent, but selective survival of non-*Haemonchus* genera or survival of mixed helminth populations was also observed (see Supplementary Materials).

**Figure 5:**
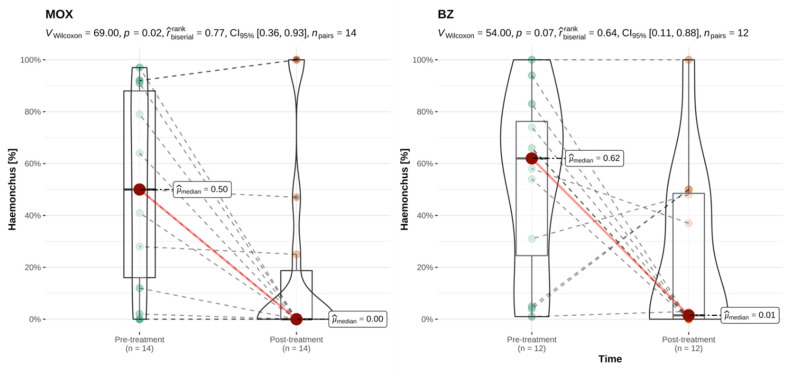
Paired comparison of the pre- and post-treatment percentage of *H. contortus* eggs for treatments performed in goats, by anthelmintic category. This analysis included all caprine treatments irrespective of their classification as successful or unsuccessful. MOX = moxidectin; BZ = benzimidazoles; *n* = number of flocks treated per category with available paired results. r^rank^_biserial_ = effect size with CI_95%_ = 95% confidence interval; *n*_pairs_ = number of paired results. Interpretation of r^rank^_biserial_: <0.05: tiny; ≥0.05 and < 0.1: very small; ≥0.1 and <0.2: small; ≥0.2 and <0.3: medium; ≥0.3 and <0.4: large; ≥0.4: very large [45].

**Figure 6:**
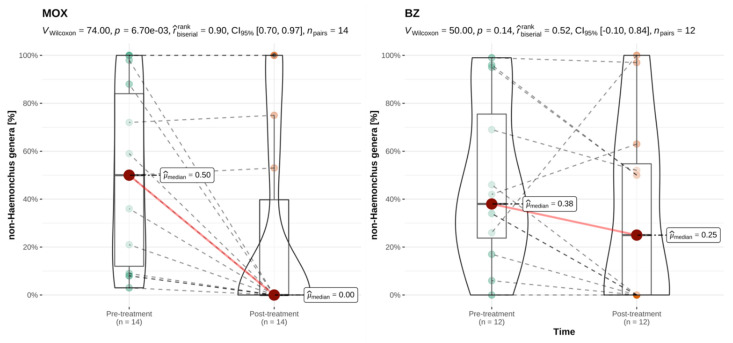
Paired comparison of the pre- and post-treatment percentage of non-*Haemonchus* strongyle eggs for treatments performed in goats, by anthelmintic category. This analysis included all caprine treatments irrespective of their classification as successful or unsuccessful. MOX = moxidectin; BZ = benzimidazoles; *n* = number of flocks treated per category with available paired results. r^rank^_biserial_ = effect size with CI_95%_ = 95% confidence interval; *n*_pairs_ = number of paired results. Interpretation of r^rank^_biserial_: < 0.05: tiny; ≥0.05 and <0.1: very small; ≥0.1 and <0.2: small; ≥0.2 and <0.3: medium; ≥0.3 and <0.4: large; ≥0.4: very large [45]. Explanation of scientific notation for *p*-values: 6.70e-03 is equivalent to *p* = 0.007.

Since levamisole, avermectins, and monepantel were each used in only three goat herds, potential selective effectiveness of these treatments against *H. contortus* or non-*Haemonchus* genera could not be reliably assessed for this species. The closantel and mebendazole combination product was not used in the examined goat herds. The results for moxidectin and benzimidazoles and the two strongyle categories are shown in Figure 5 and Figure 6. In the examined goat herds, treatments with moxidectin and benzimidazoles led to a significant reduction of the percentage of *H. contortus* eggs. However, the percentage of non-*Haemonchus* eggs was only significantly reduced following treatments with moxidectin. The survival of either or both helminth categories was, however, also seen following caprine treatments with these anthelmintic classes (see Supplementary Materials).

The predominant type (≥90% of eggs counted) of strongyle eggs present in post-treatment samples for ovine treatments classified as unsuccessful (FECR < 95%) was identified as *H. contortus* in 23 of 39 cases for moxidectin (≥90% other strongyle genera [other]: 11/39; mixed: 5/39), 0 out of 3 for avermectins (other: 0/0; mixed: 3/3), 1 out of 6 for levamisole (other: 5/6; mixed: 0/6), 1 out of 3 for monepantel (other: 0/3; mixed: 2/3), 10 out of 23 for benzimidazoles (other: 2/23; mixed: 11/23), and 0 out of 2 for the closantel and mebendazole combination (other: 2/2; mixed: 0/2). For unsuccessful treatments in goats, *H. contortus* was the predominant type of strongyle eggs in post-treatment samples for 2 out of 3 treatments with moxidectin (other: 0/3; mixed: 1/3), 1 out of 3 for avermectins (other: 0/3; mixed: 2/3), 0 out of 3 for levamisole (other: 3/3; mixed: 0/3), 0 out of 1 for monepantel (other: 1/1; mixed: 0/1), and 1 out of 6 for benzimidazoles (other: 1/6; mixed: 4/6).

Of the 25 farmers (sheep: 21; goats: 4) using more than one anthelmintic class in their respective animal groups, ineffectiveness of two anthelmintic classes was seen in three ovine cases (2× moxidectin and benzimidazoles; 1× moxidectin and closantel and mebendazole), and of all three applied anthelmintic classes (benzimidazoles, levamisole, and moxidectin) in one ovine case.

## 4. Discussion

The level of reduced anthelmintic efficacy in Europe is highly concerning [4,5], and multi-species resistance [46,47] and multi-drug resistance [48,49,50,51] are also increasingly being reported. The current situation in Germany is, however, very poorly studied to date. Previous small-scale studies [7,8,9,11] or individual case reports [10,12] do not reflect the true picture regarding the effectiveness of performed anthelmintic treatments in the field. This study was able to include a large number of farms and to simultaneously assess treatments using all anthelmintic classes available in one country in both small ruminant species. It provides additional information regarding effectiveness of performed treatments by incorporating the information obtained by differentiating *H. contortus* and non-*Haemonchus* genera to assess potential selective survival, rather than relying on FECR alone.

In order to reach the broadest possible participation, some compromises had to be made in methodology, as performance of the classical FECRT [29] is impracticable for large-scale field studies [31,38,39], and an optimal degree of standardization cannot be achieved in studies relying on farmer participation. Simplified protocols were therefore necessary to gain access to sufficient farm numbers representing the variety of the target population and to gain a realistic impression of the current situation regarding the effectiveness of treatments performed in the field. Farmer compliance and cost-effectiveness were crucial for the recruitment of sufficient farm numbers eligible for and willing to participate in post-treatment examinations. A simplified composite sampling approach and a modified McMaster technique were therefore chosen for these reasons.

The methods used in this study thus deviate in several aspects from current recommendations regarding the performance of a FECRT for the assessment of anthelmintic resistance [26], and results need to be interpreted with caution with regard to conclusions being drawn regarding anthelmintic efficacy. The latest recommendations for the performance of a FECRT include a protocol based on the examination of composite samples but require pooling of the samples in the laboratory [26]. While farmers were asked to add similar amounts of faeces from each animal to the sample container, this level of accuracy could not be achieved in the present study. In addition, the sampling and treatment procedures could not be directly overseen by the authors due to logistical constraints. Detailed instructions were provided to the participants, but adherence to these remains a matter of trust. Potential uncertainties thus involve sampling technique, animal selection and identity, animal weights, calibration of dosing equipment, dosing technique, storage of anthelmintic compounds and correctness of information provided in the questionnaire. However, many samples were submitted by veterinarians rather than the farmers themselves, thus ensuring a certain level of veterinary supervision in these cases, and the status of anthelmintics as prescription-only drugs in Germany generally requires veterinary advice upon prescription. Farmers also have a vital interest in performing effective treatments for the sake of health, wellbeing, and productivity of their animals, but uncertainties remain.

Latest recommendations for the performance of a FECRT include a minimum crude egg count of 200 from an examined animal group [26] to ensure adequate assessment of FECR. These latest recommendations were not yet available when this study was initiated. Methods were therefore defined according to the available literature at the time, also taking into account cost and practicability to allow the examination of large sample numbers. Coles et al. (1992) recommend an inclusion threshold of a group mean egg count > 150 epg determined from the examination of individual samples [29]. Since mean values of individually determined egg counts have been shown to correlate well with pooled samples, particularly for pools of five as used in this study [38], the required pool size was set at five animals per pool, and an inclusion threshold of >200 epg (exceeding the recommendations of Coles et al. (1992) [29] on the basis of caution) was chosen for the examined animal groups. While the use of pooled samples has been shown to correlate well with individually determined results for the assessment of FEC, FECR, and classification of treatment success [38,39,52], careful consideration needs to be given to pool size, baseline egg counts, and the analytical sensitivity of the method used. Modified McMaster methods, albeit less sensitive than Mini-FLOTAC in the detection of low egg counts, have been shown to provide similarly reliable results in the assessment of FECR and anthelmintic efficacy from pooled samples, particularly when baseline egg counts are relatively high. The Mini-FLOTAC technique is however preferable for samples containing very low egg numbers [38,39]. The use of pooled samples and a modified McMaster technique with intermediate analytical sensitivity as used in this study may thus contribute to erroneous negative egg counts for samples containing low quantities of strongyle eggs, and thus potentially over-estimate the effectiveness of the performed treatments [39]. Another limitation of a composite FECRT approach is that calculation of 95% confidence intervals is not possible [52]. These are however required for precise assessment and thus essentially proof of anthelmintic resistance [26,29]. It is thus recommended that FECR results based on composite samples should be interpreted with caution, suggesting that results ≥ 95% can be interpreted as successful, while FECR ≤ 80% can be reasonably associated with anthelmintic resistance [52]. For values between 80 and 95% there remains some level of uncertainty [52], but resistance in considered likely for values < 90% [26]. Composite samples also essentially provide an estimated mean egg count of the animals contributing to the pool and thus do not allow identification of individual high-shedding animals [52]. They are thus not suitable for targeted selective treatment approaches and only provide information on the infection density of animal groups.

The initial study design of pre- and post-treatment examination of three pools each representing five individual animals, thus including a total of 15 individuals per treatment, was counter-acted by a number of farmers who chose to treat their different animal groups with different anthelmintics. In addition, adherence to the inclusion threshold of initial egg excretion > 200 epg did sometimes not allow inclusion of all three initially screened management groups per farm. While 57.7% of the treatment assessments were based on ten or 15 animals, a considerable percentage of the treatment outcomes could only be assessed based on one sample pool, representing five individual animals. For the performance of a FECRT for assessment of anthelmintic resistance, ten to 15 animals need to be sampled [26,29,31]. Treatment assessments based on less than ten animals may be less accurate and potentially over-estimate treatment effectiveness, particularly if initial egg counts are low. In conjunction with changes in pre-and post-treatment *H. contortus* percentage and, in many cases, high initial egg counts, we believe that they do provide important information regarding treatment effectiveness, and these assessments were therefore retained in the study.

Since treatments were chosen by the participants and the authors could not anticipate which anthelmintic class would be used, farmers were asked to adhere to a convenience treatment-to-sampling interval as recommended for the concurrent evaluation of several anthelmintic classes [29,30] to avoid too complicated or confusing instructions. This convenience approach has also been used in previous published studies, including levamisole [39,53], for which a shorter optimal treatment-to-sampling interval is usually recommended because it is less effective against inhibited larval stages [30]. Optimal treatment-to-sampling intervals vary between anthelmintic classes, and 7–10 days are recommended for levamisole, 10–14 days for benzimidazoles, 14–17 days for avermectins and 17–21 days for moxidectin [31]. Kaplan (2020) however suggests a treatment-to-sampling interval of 10–14 days for levamisole as well as benzimidazoles [26]. No recommendations have been published for the use of monepantel or closantel. Simultaneous sampling at 14 days post treatment is recommended if more than one anthelmintic class is tested at the same time [26,30]. The accepted treatment-to-sampling intervals for the different anthelmintic classes in this study were wider than these recommendations as the natural variation entailed with sample collection by farmers had to be accounted for. They were however adjusted to the individual properties of the different anthelmintic classes by removal of outliers for the given anthelmintic category. In case of levamisole, the use of a longer than optimal treatment-to-sampling interval may be associated with under-estimation of treatment effectiveness. Benzimidazoles and macrocyclic lactones can cause temporary inhibition of egg excretion [30], the use of a shorter than optimal treatment-to-sampling interval may thus be associated with over-estimation of treatment effectiveness.

There is also likely to be a degree of potential over-estimation of treatment success inherent to the laboratory and sampling methods used in this study as discussed above. Over-estimation of treatment success can also be associated with the applied threshold of ≥95% FECR, as FECR results are also influenced by species composition of the treated helminth population. Low levels of anthelmintic resistance present in a flock in terms of a low abundance of helminths carrying resistance alleles, or low fecundity of resistant worm species compared to their non-resistant counterparts within a helminth population can lead to FECR ≥ 95% even though resistant nematodes may be present, and thus mask the existence of early resistance in a flock [40,54]. On the other hand, under-estimation of efficacy is also possible in our study due to the discussed uncertainties regarding farmer-performed treatments. Amongst other factors, application errors or under-estimation of animal weights, and thus involuntary underdosing, can lead to over-estimation of treatment ineffectiveness by incorrectly attributing treatment failure to other factors such as suspectedly reduced drug efficacy rather than human error [55].

Despite the discussed uncertainties and the use of field data, we believe that the observed effectiveness of anthelmintic treatments is largely driven by anthelmintic efficacy in our study population, as best possible efforts were made under the circumstances to ensure correct sample collection and treatments, reliable laboratory techniques, careful selection of participating flocks based on sufficiently high baseline egg counts, and exclusion of participants not adhering to the study requirements. This assumption is also supported by highly significant differences between the anthelmintic classes, as confounding factors and methodical limitations equally apply to all performed treatments, and by the observed changes in percentages of *H. contortus*- and non-*Haemonchus* eggs between pre- and post-treatment samples, indicating selective survival of either *H. contortus* or non-*Haemonchus* genera in many treated animal groups (see Supplementary Materials). The observed reduction in pre- and post-treatment egg counts frequently fell below the threshold defined as indicative for “likely” or “highly likely” presence of anthelmintic resistance [26]. There were significant differences between the different anthelmintic classes regarding predicted probabilities of FECR, with benzimidazoles showing the lowest performance, followed by moxidectin, a drug that has long been considered one of the most potent anthelmintic compounds [24] and which has been heavily relied on in the past. Ineffective treatments were also observed for the most recently detected anthelmintic compound available to the participants, monepantel, and this has not previously been reported in small ruminants in Germany. Closantel was only used as part of a combination product with mebendazole. This narrow-spectrum anthelmintic selectively active against *H. contortus* could therefore not be assessed individually. Fluorescence staining of strongyle eggs however revealed that the percentage of *H. contortus* eggs was reduced to zero in all flocks treated with this product. The effectiveness of closantel-based treatments against its target species was thus considered highly satisfactory in the studied flocks. Eggs present in post-treatment samples following application of this combination product were all identified as non-*Haemonchus* genera and are thus most likely associated with ineffectiveness of the benzimidazole compound.

While all examined anthelmintic classes except the closantel and mebendazole combination achieved a significant reduction in the percentage of non-*Haemonchus* eggs across all examined sheep flocks, only treatments with levamisole, monepantel, and the closantel and mebendazole combination were associated with a significant reduction in *H. contortus* percentage. This parasite thus seems to be widely responsible for the observed treatment failures for moxidectin and benzimidazoles. More detailed, molecular studies on the pre- and post-treatment nematode populations [36,37,47] are a matter for future research in order to more accurately assess the species composition, and to identify potentially resistant helminth species.

The low number of treatments in goats hampered separate statistical analyses for this species. Differences between the two small ruminant species could therefore not be adequately assessed. However, it is interesting to note that 25% (10/40) of the initially participating caprine herds had to be excluded from further analyses due to participants not using appropriately increased dose rates for this species. There thus still seem to be knowledge gaps in the veterinary and farming community regarding adequate dose rates for goats treated under cascade regulations. This frequency of underdosing in goats is concerning, particularly in light of goats frequently being seen as drivers of anthelmintic resistance [56], and further educational efforts are required to ensure adequate treatment of this species.

The recent licensing of eprinomectin as currently the only anthelmintic product for use in goats in Germany is on the one hand a positive development as it ensures detailed dose response trials [20,57,58] and removes uncertainties regarding adequate dose rates for this species, it however limits the legal treatment options for goats to the use of a single anthelmintic compound unless it has already proven ineffective, and thus poses a dilemma for the application of modern recommendations regarding responsible use of anthelmintics, which, amongst other measures, require rotational use of different anthelmintic classes to slow the selection of resistance [25]. Similar concerns have also been previously raised by Rostang et al. (2020) [59], and its use as a pour-on product has also proven controversial [20,59]. Widespread use of a single product in dairy goats in Switzerland has been previously shown to select for unsustainably high levels of avermectin resistance [60]. There is also only a very small selection of licensed anthelmintic compounds for use in dairy sheep in Germany, currently limited to benzimidazoles and macrocyclic lactones, and the already highly concerning situation regarding treatment effectiveness following use of these compounds is likely to worsen considerably within the near future if these shortcomings are not addressed by pharmaceutical companies and licensing authorities.

## 5. Conclusions

While the study design did not allow for conclusive proof of anthelmintic resistance, the observed proportion of ineffective treatments in small ruminant flocks in Germany is worrying and highly likely to be influenced by reduced efficacy of anthelmintic compounds. The results are particularly concerning for benzimidazoles and macrocyclic lactones, but ineffective treatments were observed for all currently available anthelmintic classes including the most recently discovered anthelmintic compound available in Germany, monepantel. Further work involving molecular analyses and follow-up visits to participating farms is desirable to complement the results of this field study and to confirm suspected anthelmintic resistance. A change in attitudes regarding parasite management and towards sustainable helminth control is urgently needed to prevent rapid selection of anthelmintic resistance in German small ruminant flocks and further afield.

## Figures and Tables

**Table 1:** Anthelmintics used by farmers and their veterinarians in 223 small ruminant flocks which fulfilled the inclusion criteria for post-treatment assessment of treatment effectiveness, specifying the number of sheep flocks/goat herds in each anthelmintic category. Some farmers used different anthelmintics in different animal groups, a total of 253 treatments were thus performed.

Anthelmintic Compound/Class	Number of Sheep Flocks	Number of Goat Herds
Moxidectin ^1^	86	14
Avermectins ^2^	5	3
Levamisole ^3^	41	3
Monepantel ^1^	25	3
Benzimidazoles ^1^	44	12
Closantel and mebendazole ^1^	17	0
(Combination product)		

^1^ oral preparations only; ^2^ injectable preparations with the exception of one caprine herd treated with pour-on product; ^3^ oral preparations with the exception of one ovine flock treated with injectable product.

**Table 2:** Number of flocks (both small ruminant species) with suspected previous ineffective treatments as stated in the questionnaire, by anthelmintic category, and number of flocks using the suspected product for this study, including respective treatment success. Ineffectiveness was suspected for multiple compounds/classes by some participants. *n* = 72 flocks reporting 92 suspected previous treatment failures.

Anthelmintic Compound/Class (Number of Sheep Flocks/Goat Herds Suspecting Previous Ineffectiveness)	Number of These Flocks (Sheep/Goats) Using Suspected Product	Number of Flocks (Sheep/Goats) with Unsuccessful Treatment(s) Using Suspected Product
Moxidectin ^1^ (33/3)	12/2	6/0
Avermectins ^2^ (6/3)	1/0	0/n.a.
Levamisole ^3^ (6/1)	3/0	0/n.a.
Monepantel ^1^ (8/1)	2/0	1/n.a.
Benzimidazoles ^1^ (24/3)	5/2	0/2
Closantel and mebendazole ^1^ (3/1)	0/0	n.a./n.a.
(Combination product)		

^1^ oral preparations only; ^2^ injectable preparations with the exception of one caprine herd treated with pour-on product; ^3^ oral preparations with the exception of one ovine flock treated with injectable product; n.a. = not applicable.

**Table 3:** Proportion and percentage of unsuccessful treatments (faecal egg count reduction [FECR] < 95%) in 223 small ruminant flocks performing 253 treatments, by anthelmintic compound/class. Treatments chosen and performed by farmers and their veterinarians. Pooled samples were used to assess FECR, and some farmers used different anthelmintics in different animal groups.

Anthelmintic Compound/Class (Number of Sheep Flocks/Goat Herds Using These)	Number and Percentage of Flocks with Unsuccessful Treatment(s)	
	Sheep	Goats
Moxidectin ^1^ (86/14)	39 (45.3%)	3 (21.4%)
Avermectins ^2^ (5/3)	3 (60.0%)	3 (100%)
Levamisole ^3^ (41/3)	6 (14.6%)	3 (100%)
Monepantel ^1^ (25/3)	3 (12.0%)	1 (33.3%)
Benzimidazoles ^1^ (44/12)	23 (52.3%)	6 (50.0%)
Closantel and mebendazole ^1^ (17/0)	2 (11.8%)	n.a.
(Combination product)		

^1^ oral preparations only; ^2^ injectable preparations with the exception of one caprine herd treated with pour-on product; ^3^ oral preparations with the exception of one ovine flock treated with injectable product; n.a. = not applicable.

**Table 4:** Descriptive statistics of faecal egg count reduction (FECR) for all treatments (both small ruminant species, and including successful and unsuccessful treatments), by anthelmintic category. Treatments chosen and performed by farmers and their veterinarians. Pooled samples were used, and the samples originated from 223 small ruminant flocks, which performed 253 treatments. SD = standard deviation; IQR = inter quartile range; *n* = number of flocks using anthelmintic category.

Anthelmintic Compound/Class (Number of Sheep Flocks/Goat Herds Using These)	Mean FECR (Sheep/Goats)	SD (Sheep/Goats)	Median FECR (Sheep/Goats)	IQR (Sheep/Goats)
Moxidectin ^1^ (86/14)	81.8%/94.3%	27.8/11.4	95%/100%	21.5/4.5
Avermectins ^2^ (5/3)	56.6%/90.0%	52.1/1.7	83%/89%	100/1.5
Levamisole ^3^ (41/3)	97.7%/87.3%	3.9/1.2	100%/88%	3.0/1.0
Monepantel ^1^ (25/3)	97.4%/90.0%	7.2/15.6	100%/98%	0.0/1.4
Benzimidazoles ^1^ (44/12)	66.9%/78.3%	39.9/34.6	93%/96%	59.3/30.0
Closantel and mebendazole ^1^ (17/0)	94.0%/n.a.	15.4/n.a.	100%/n.a.	1.0/n.a.
(Combination product)				

^1^ oral preparations only; ^2^ injectable preparations with the exception of one caprine herd treated with pour-on product; ^3^ oral preparations with the exception of one ovine flock treated with injectable product; n.a. = not applicable.

**Table 5:** Results of pairwise comparisons of the examined anthelmintic categories regarding predicted probabilities of faecal egg count reduction in sheep. MOX = moxidectin; AV = avermectins, LEV = levamisole; MOP = monepantel; BZ = benzimidazoles; CLOS + BZ = closantel and mebendazole; OR = odds ratio; 95% CI = 95% confidence interval. Significant results (*p ≤* 0.05) and tendencies (*p* > 0.05 and <0.1) and are both indicated in bold.

Paired Comparison	OR [95% CI]	*p*-Value (Following Holm Correction)
MOX/AV	3.45 [0.55–21.81]	0.292
MOX/LEV	**0.10 [0.01–0.87]**	**0.019**
MOX/MOP	**0.12 [0.01–1.48]**	**0.093**
MOX/BZ	**2.22 [0.97–5.10]**	**0.042**
MOX/CLOS + BZ	0.29 [0.04–2.27]	0.379
AV/LEV	**0.03 [0.00–0.45]**	**0.002**
AV/MOP	**0.03 [0.00–0.71]**	**0.013**
AV/BZ	0.64 [0.10–4.18]	1.000
AV/CLOS + BZ	**0.08 [0.01–1.19]**	**0.048**
LEV/MOP	1.15 [0.05–28.08]	1.000
LEV/BZ	**21.29 [2.50–181.06]**	**<0.001**
LEV/CLOS + BZ	2.73 [0.16–47.78]	1.000
MOP/BZ	**18.51 [1.47–232.71]**	**0.009**
MOP/CLOS + BZ	2.38 [0.10–56.26]	1.000
BZ/CLOS + BZ	**0.13 [0.02–1.04]**	**0.040**

## Data Availability

Data presented in this study are available in the Supplementary Materials file. Further information can be obtained from the corresponding author upon reasonable request.

## Supplementary Materials

The following supporting information can be downloaded at: https://www.mdpi.com/article/10.3390/ani12121501/s1, Table S1: Detailed results of treatment assessments.
