# Supplementary material for: Effectiveness of Anthelmintic Treatments in Small Ruminants in Germany
Source: Animals (Basel). 2022 Jun 9;12(12):1501. doi: 10.3390/ani12121501 (PMC9219448; doi:10.3390/ani12121501)
Supplement: Supplementary file 1 [file animals-12-01501-s001.zip › animals-1739372-supplementary.pdf]

Supplementary Table S1. Detailed results for the individual treatments (n=253, from 223 small ruminant flocks) specifying relevant parameters: small ruminant species, pre- and post-treatment percentage of *H. contortus* eggs (identified by PNA-FITC stain) and of other strongyle eggs, pre- and post-treatment faecal egg counts, reduction between pre- and post-treatment egg counts (FECR), treatment success (FECR ≥95%), applied anthelmintic compound, dose rate and route of application, number of animals contributing to the results and treatment to sampling interval. Faecal egg counts (FEC) below the detection limit of the modified McMaster method (< 33.3 epg) are given as zero. FEC and percentages of *H. contortus* / other strongyle eggs are presented as the arithmetic mean of the examined samples per treatment (5 animals: 1 pooled sample of five; 10 animals: 2 pooled samples of five; 15 animals: 3 pooled samples of five). Flock numbers indicated in red used more than one anthelmintic compound in different animal groups, these treatments are listed separately under the respective anthelmintic compound.

Flock ID	Species	Pre-treatment *Haemonchus* eggs (%)	Pre-treatment other strongyle eggs (%)	Post-treatment *Haemonchus* eggs (%)	Post-treatment other strongyle eggs (%)	Pre-treatment FEC (epg)	Post-treatment FEC (epg)	FECR (%)	Treatment success	Anthelmintic compound	Dose	Route of applica-tion	Number animals	Treatment to sampling interval (days)
**MS1**	sheep	31	69	0	0	1000	0	100	yes	Moxidectin	licensed dose	oral	5	13
**MS103**	sheep	65	35	100	0	1300	100	92	no	Moxidectin	licensed dose	oral	5	12
**MS111**	sheep	65	35	0	0	500	0	100	yes	Moxidectin	licensed dose	oral	5	10
**MS112**	sheep	08	92	4	96	889	400	55	no	Moxidectin	licensed dose	oral	15	10
**MS117**	sheep	93	07	100	0	567	756	0	no	Moxidectin	licensed dose	oral	15	12
**MS12**	sheep	84	16	0	0	1633	0	100	yes	Moxidectin	licensed dose	oral	5	13
**MS120**	sheep	18	02	0	0	600	0	100	yes	Moxidectin	licensed dose	oral	5	15
**MS122**	sheep	27	73	0	0	1033	0	100	yes	Moxidectin	licensed dose	oral	10	13
**MS124**	sheep	0	100	0	0	400	0	100	yes	Moxidectin	licensed dose	oral	5	15
**MS128**	sheep	83	17	94	6	889	911	0	no	Moxidectin	licensed dose	oral	15	14
**MS138**	sheep	47	53	93	7	717	500	30	no	Moxidectin	licensed dose	oral	10	17
**MS139**	sheep	0	100	0	0	267	0	100	yes	Moxidectin	licensed dose	oral	10	11
**MS14**	sheep	20	80	0	100	955	155	84	no	Moxidectin	licensed dose	oral	15	13
**MS143**	sheep	0	100	0	100	467	133	72	no	Moxidectin	licensed dose	oral	5	11
**MS144**	sheep	78	22	0	0	533	0	100	yes	Moxidectin	licensed dose	oral	5	21
**MS146**	sheep	97	3	80	20	1200	133	89	no	Moxidectin	licensed dose	oral	5	12
**MS147**	sheep	6	94	0	0	400	0	100	yes	Moxidectin	licensed dose	oral	10	13
**MS149**	sheep	23	77	0	100	484	34	93	no	Moxidectin	licensed dose	oral	10	13

Flock ID	Species	Pre-treatment *Haemonchus* eggs (%)	Pre-treatment other strongyle eggs (%)	Post-treatment *Haemonchus* eggs (%)	Post-treatment other strongyle eggs (%)	Pre-treatment FEC (epg)	Post-treatment FEC (epg)	FECR (%)	Treatment success	Anthelmintic compound	Dose	Route of applica-tion	Number animals	Treatment to sampling interval (days)
**MS154**	sheep	96	4	0	0	9467	0	100	yes	Moxidectin	licensed dose	oral	5	14
**MS163**	sheep	94	6	0	0	900	0	100	yes	Moxidectin	licensed dose	oral	5	10
**MS166**	sheep	8	92	0	100	333	67	80	no	Moxidectin	licensed dose	oral	10	13
**MS167**	sheep	91	9	100	0	367	100	73	no	Moxidectin	licensed dose	oral	5	13
**MS174**	sheep	92	8	100	0	867	400	54	no	Moxidectin	licensed dose	oral	5	17
**MS175**	sheep	96	4	100	0	967	767	21	no	Moxidectin	licensed dose	oral	10	16
**MS177**	sheep	12	88	0	0	2433	0	100	yes	Moxidectin	licensed dose	oral	5	11
**MS179**	sheep	12	88	0	100	417	50	88	no	Moxidectin	licensed dose	oral	10	10
**MS181**	sheep	45	55	0	0	1517	0	100	yes	Moxidectin	licensed dose	oral	10	11
**MS185**	sheep	73	27	100	0	400	67	83	no	Moxidectin	licensed dose	oral	5	12
**MS188**	sheep	64	36	83	17	2384	117	95	yes	Moxidectin	licensed dose	oral	10	13
**MS190**	sheep	85	15	100	0	533	267	50	no	Moxidectin	licensed dose	oral	5	13
**MS193**	sheep	0	100	0	0	267	0	100	yes	Moxidectin	licensed dose	oral	5	14
**MS197**	sheep	21	79	100	0	1033	17	98	yes	Moxidectin	licensed dose	oral	10	12
**MS199**	sheep	0	100	0	0	1067	0	100	yes	Moxidectin	licensed dose	oral	5	11
**MS204**	sheep	3	97	0	0	400	0	100	yes	Moxidectin	licensed dose	oral	5	11
**MS210**	sheep	1	99	0	0	767	0	100	yes	Moxidectin	licensed dose	oral	10	10
**MS43**	sheep	27	73	99	1	1366	322	76	no	Moxidectin	licensed dose	oral	15	13
**MS45**	sheep	12	88	0	100	633	89	86	no	Moxidectin	licensed dose	oral	15	19
**MS47**	sheep	0	100	0	0	400	0	100	yes	Moxidectin	licensed dose	oral	5	12
**MS54**	sheep	11	89	100	0	700	33	95	yes	Moxidectin	licensed dose	oral	5	12
**MS56**	sheep	0	100	0	100	517	34	93	no	Moxidectin	licensed dose	oral	10	13
**MS57**	sheep	35	65	0	100	233	33	86	no	Moxidectin	licensed dose	oral	5	13
**MS64**	sheep	100	0	100	0	1056	1187	0	no	Moxidectin	licensed dose	oral	15	13
**MS80**	sheep	31	69	89	11	1433	145	90	no	Moxidectin	licensed dose	oral	15	15
**MS81**	sheep	35	65	100	0	1000	33	97	yes	Moxidectin	licensed dose	oral	5	10

Flock ID	Species	Pre-treatment *Haemonchus* eggs (%)	Pre-treatment other strongyle eggs (%)	Post-treatment *Haemonchus* eggs (%)	Post-treatment other strongyle eggs (%)	Pre-treatment FEC (epg)	Post-treatment FEC (epg)	FECR (%)	Treatment success	Anthelmintic compound	Dose	Route of applica-tion	Number animals	Treatment to sampling interval (days)
**MS82**	sheep	38	62	50	50	600	33	95	yes	Moxidectin	licensed dose	oral	10	12
**MS87**	sheep	93	07	100	0	1400	684	51	no	Moxidectin	licensed dose	oral	10	12
**MS89**	sheep	62	38	0	0	676	0	100	yes	Moxidectin	licensed dose	oral	5	13
**MS90**	sheep	81	19	98	2	1533	1067	30	no	Moxidectin	licensed dose	oral	5	11
**MS92**	sheep	90	10	96	4	3683	1400	62	no	Moxidectin	licensed dose	oral	10	14
**MS95**	sheep	97	3	0	0	2033	0	100	yes	Moxidectin	licensed dose	oral	5	14
**S106**	sheep	14	86	100	0	2778	2156	22	no	Moxidectin	licensed dose	oral	15	12
**S108**	sheep	70	30	100	0	600	133	78	no	Moxidectin	licensed dose	oral	5	11
**S110**	sheep	35	65	100	0	1533	133	91	no	Moxidectin	licensed dose	oral	10	15
**S116**	sheep	/	/	0	0	333	0	100	yes	Moxidectin	licensed dose	oral	5	13
**S118**	sheep	58	42	100	0	1722	56	97	yes	Moxidectin	licensed dose	oral	15	14
**S121**	sheep	04	96	0	0	411	0	100	yes	Moxidectin	licensed dose	oral	15	16
**S131**	sheep	90	10	100	0	767	478	38	no	Moxidectin	licensed dose	oral	15	15
**S148**	sheep	58	42	100	0	1500	178	88	no	Moxidectin	licensed dose	oral	15	12
**S150**	sheep	89	11	0	0	21933	0	100	yes	Moxidectin	licensed dose	oral	5	10
**S151**	sheep	97	3	96	4	2466	483	80	no	Moxidectin	licensed dose	oral	10	13
**S153**	sheep	53	47	80	20	1256	22	98	yes	Moxidectin	licensed dose	oral	15	13
**S162**	sheep	7	93	72	28	267	400	0	no	Moxidectin	licensed dose	oral	5	13
**S178**	sheep	30	70	92	8	2500	200	92	no	Moxidectin	licensed dose	oral	5	10
**S185**	sheep	65	35	0	0	2900	0	100	yes	Moxidectin	licensed dose	oral	10	16
**S189**	sheep	9	91	0	0	400	0	100	yes	Moxidectin	licensed dose	oral	15	11
**S190**	sheep	5	95	0	0	333	0	100	yes	Moxidectin	licensed dose	oral	15	11
**S195**	sheep	58	42	48	52	1017	500	51	no	Moxidectin	licensed dose	oral	10	14
**S200**	sheep	13	87	100	0	700	484	31	no	Moxidectin	licensed dose	oral	10	17
**S202**	sheep	17	83	0	100	417	17	96	yes	Moxidectin	licensed dose	oral	10	12
**S203**	sheep	0	100	0	0	267	0	100	yes	Moxidectin	licensed dose	oral	5	13

Flock ID	Species	Pre-treatment *Haemonchus* eggs (%)	Pre-treatment other strongyle eggs (%)	Post-treatment *Haemonchus* eggs (%)	Post-treatment other strongyle eggs (%)	Pre-treatment FEC (epg)	Post-treatment FEC (epg)	FECR (%)	Treatment success	Anthelmintic compound	Dose	Route of applica-tion	Number animals	Treatment to sampling interval (days)
**S207**	sheep	96	4	60	40	1967	100	95	yes	Moxidectin	licensed dose	oral	5	14
**S215**	sheep	0	100	0	0	467	0	100	yes	Moxidectin	licensed dose	oral	5	13
**S219**	sheep	11	89	0	100	833	17	98	yes	Moxidectin	licensed dose	oral	10	12
**S221**	sheep	13	87	11	89	467	44	91	no	Moxidectin	licensed dose	oral	15	10
**S223**	sheep	67	33	100	0	2467	100	96	yes	Moxidectin	licensed dose	oral	10	14
**S227**	sheep	82	18	100	0	233	40	81	no	Moxidectin	licensed dose	oral	15	10
**S232**	sheep	1	99	0	0	2500	0	100	yes	Moxidectin	licensed dose	oral	5	10
**S32**	sheep	4	96	0	0	383	0	100	yes	Moxidectin	licensed dose	oral	10	12
**S43**	sheep	0	100	0	100	300	167	44	no	Moxidectin	licensed dose	oral	5	11
**S46**	sheep	8	92	0	100	1156	56	95	yes	Moxidectin	licensed dose	oral	15	14
**S56**	sheep	/	/	0	0	300	0	100	yes	Moxidectin	licensed dose	oral	5	13
**S66**	sheep	72	28	0	0	1167	0	100	yes	Moxidectin	licensed dose	oral	5	14
**S69**	sheep	16	84	0	0	900	0	100	yes	Moxidectin	licensed dose	oral	10	13
**S79**	sheep	0	100	0	100	434	167	62	no	Moxidectin	**2.5x licensed dose**	oral	10	15
**S85**	sheep	13	87	1	0	633	22	97	yes	Moxidectin	licensed dose	oral	15	11
**S88**	sheep	17	83	0	50	967	17	98	yes	Moxidectin	licensed dose	oral	10	13
**MS21**	sheep	1	99	0	0	356	0	100	yes	Doramectin	licensed dose	injection	15	13
**MS30**	sheep	33	67	86	14	900	150	83	no	Doramectin	licensed dose	injection	10	13
**MS58**	sheep	0	100	0	0	933	0	100	yes	Ivermectin	**1,8x licensed dose**	injection	5	13
**MS59**	sheep	29	71	45	55	922	1189	0	no	Doramectin	licensed dose	injection	15	11
**MS75**	sheep	15	85	61	39	484	583	0	no	Doramectin	licensed dose	injection	10	12
**MS101**	sheep	13	87	0	0	350	0	100	yes	Levamisol	licensed dose	oral	10	10
**MS111**	sheep	18	82	0	0	334	0	100	yes	Levamisol	licensed dose	oral	10	10
**MS116**	sheep	31	69	0	0	2400	0	100	yes	Levamisol	licensed dose	oral	5	12
**MS123**	sheep	17	83	0	0	267	0	100	yes	Levamisol	licensed dose	oral	15	7

Flock ID	Species	Pre-treatment *Haemonchus* eggs (%)	Pre-treatment other strongyle eggs (%)	Post-treatment *Haemonchus* eggs (%)	Post-treatment other strongyle eggs (%)	Pre-treatment FEC (epg)	Post-treatment FEC (epg)	FECR (%)	Treatment success	Anthelmintic compound	Dose	Route of application	Number animals	Treatment to sampling interval (days)
MS124	sheep	0	100	0	0	300	0	100	yes	Levamisol	licensed dose	oral	5	15
MS127	sheep	0	100	0	0	600	0	100	yes	Levamisol	licensed dose	oral	5	10
MS132	sheep	10	90	8	92	622	55	91	no	Levamisol	licensed dose	oral	15	14
MS161	sheep	66	34	0	100	2500	33	99	yes	Levamisol	licensed dose	oral	5	14
MS167	sheep	77	23	100	0	267	33	88	no	Levamisol	licensed dose	oral	5	13
MS170	sheep	13	87	0	100	689	11	98	yes	Levamisol	licensed dose	oral	15	7
MS173	sheep	10	90	0	0	1867	0	100	yes	Levamisol	licensed dose	oral	5	11
MS182	sheep	88	12	0	0	233	0	100	yes	Levamisol	licensed dose	oral	5	12
MS190	sheep	42	58	0	0	333	0	100	yes	Levamisol	licensed dose	oral	5	13
MS47	sheep	0	100	0	100	800	67	92	no	Levamisol	licensed dose	oral	5	15
MS48	sheep	0	100	0	100	317	50	84	no	Levamisol	licensed dose	oral	10	12
MS49	sheep	0	100	0	0	600	0	100	yes	Levamisol	licensed dose	oral	5	12
MS52	sheep	0	100	0	100	750	50	93	no	Levamisol	licensed dose	oral	10	14
MS58	sheep	0	100	0	100	1133	17	98	yes	Levamisol	licensed dose	oral	10	13
MS60	sheep	0	100	0	100	700	34	95	yes	Levamisol	licensed dose	oral	10	8
MS63	sheep	76	24	9	91	883	84	90	no	Levamisol	**2.5x licensed dose**	oral	10	14
MS73	sheep	5	95	0	0	500	0	100	yes	Levamisol	licensed dose	oral	5	15
MS74	sheep	94	6	0	0	5084	17	100	yes	Levamisol	licensed dose	oral	10	9
MS79	sheep	0	100	0	0	333	0	100	yes	Levamisol	licensed dose	oral	5	15
MS86	sheep	15	85	3	97	1533	78	95	yes	Levamisol	licensed dose	oral	15	16
S115	sheep	31	69	0	0	3911	0	100	yes	Levamisol	licensed dose	oral	15	8
S139	sheep	0	100	0	0	467	0	100	yes	Levamisol	licensed dose	oral	5	13
S140	sheep	/	/	0	100	900	33	96	yes	Levamisol	licensed dose	oral	5	13
S188	sheep	94	6	0	0	444	0	100	yes	Levamisol	licensed dose	oral	15	14
S217	sheep	71	29	0	0	2333	0	100	yes	Levamisol	licensed dose	oral	5	13

Flock ID	Species	Pre-treatment *Haemonchus* eggs (%)	Pre-treatment other strongyle eggs (%)	Post-treatment *Haemonchus* eggs (%)	Post-treatment other strongyle eggs (%)	Pre-treatment FEC (epg)	Post-treatment FEC (epg)	FECR (%)	Treatment success	Anthelmintic compound	Dose	Route of applica-tion	Number animals	Treatment to sampling interval (days)
**S218**	sheep	46	54	0	100	400	11	97	yes	Levamisol	licensed dose	oral	15	8
**S235**	sheep	88	12	0	0	2633	0	100	yes	Levamisol	licensed dose	oral	15	13
**S29**	sheep	0	100	0	0	244	0	100	yes	Levamisol	licensed dose	oral	15	15
**S35**	sheep	37	63	0	0	1178	0	100	yes	Levamisol	licensed dose	oral	15	16
**S43**	sheep	8	92	0	0	884	0	100	yes	Levamisol	licensed dose	oral	10	11
**S44**	sheep	11	89	0	100	617	17	97	yes	Levamisol	licensed dose	oral	10	15
**S47**	sheep	0	100	0	100	678	33	95	yes	Levamisol	licensed dose	injection	15	15
**S73**	sheep	58	42	0	0	734	0	100	yes	Levamisol	licensed dose	oral	10	12
**S74**	sheep	62	38	0	0	517	0	100	yes	Levamisol	licensed dose	oral	10	12
**S75**	sheep	0	100	0	0	267	0	100	yes	Levamisol	licensed dose	oral	5	12
**S76**	sheep	0	100	0	100	989	11	99	yes	Levamisol	licensed dose	oral	15	7
**S84**	sheep	18	82	0	0	600	0	100	yes	Levamisol	licensed dose	oral	15	10
**MS110**	sheep	52	48	0	0	3533	0	100	yes	Monepantel	licensed dose	oral	10	13
**MS133**	sheep	31	69	0	0	583	0	100	yes	Monepantel	licensed dose	oral	10	10
**MS157**	sheep	51	49	0	0	1550	0	100	yes	Monepantel	licensed dose	oral	10	11
**MS159**	sheep	32	68	0	0	1111	0	100	yes	Monepantel	licensed dose	oral	15	11
**MS169**	sheep	39	61	0	0	1444	0	100	yes	Monepantel	licensed dose	oral	15	13
**MS171**	sheep	15	85	0	0	1467	0	100	yes	Monepantel	licensed dose	oral	15	11
**MS174**	sheep	94	6	0	0	1600	0	100	yes	Monepantel	licensed dose	oral	5	17
**MS177**	sheep	35	65	35	65	1433	100	93	no	Monepantel	licensed dose	oral	10	11
**MS194Z43**	sheep	84	16	0	0	900	0	100	yes	Monepantel	licensed dose	oral	10	13
**MS207**	sheep	79	21	12	88	600	200	67	no	Monepantel	licensed dose	oral	10	11
**MS50**	sheep	/	/	0	0	267	0	100	yes	Monepantel	licensed dose	oral	5	16
**MS95**	sheep	96	4	97	3	13733	700	95	yes	Monepantel	licensed dose	oral	5	14
**S125**	sheep	81	19	100	0	2833	422	85	no	Monepantel	licensed dose	oral	15	11

Flock ID	Species	Pre-treatment *Haemonchus* eggs (%)	Pre-treatment other strongyle eggs (%)	Post-treatment *Haemonchus* eggs (%)	Post-treatment other strongyle eggs (%)	Pre-treatment FEC (epg)	Post-treatment FEC (epg)	FECR (%)	Treatment success	Anthelmintic compound	Dose	Route of applica-tion	Number animals	Treatment to sampling interval (days)
**S128**	sheep	100	0	0	0	867	0	100	yes	Monepantel	licensed dose	oral	5	13
**S165**	sheep	0	100	0	0	433	0	100	yes	Monepantel	licensed dose	oral	5	17
**S167**	sheep	2	98	50	50	244	11	95	yes	Monepantel	licensed dose	oral	15	11
**S168**	sheep	1	99	0	0	267	0	100	yes	Monepantel	licensed dose	oral	15	12
**S169**	sheep	1	99	0	0	411	0	100	yes	Monepantel	licensed dose	oral	15	11
**S170**	sheep	81	19	0	0	667	0	100	yes	Monepantel	licensed dose	oral	5	12
**S171**	sheep	26	74	0	0	300	0	100	yes	Monepantel	licensed dose	oral	10	12
**S172**	sheep	34	66	0	0	367	0	100	yes	Monepantel	licensed dose	oral	10	12
**S196**	sheep	71	29	0	0	3433	0	100	yes	Monepantel	licensed dose	oral	15	14
**S198**	sheep	15	85	0	0	900	0	100	yes	Monepantel	licensed dose	oral	5	10
**S66**	sheep	43	57	0	0	1100	0	100	yes	Monepantel	licensed dose	oral	5	14
**S98**	sheep	69	31	0	0	778	0	100	yes	Monepantel	licensed dose	oral	15	14
**MS120**	sheep	23	77	100	0	967	33	97	yes	Albendazole	licensed dose	oral	5	11
**MS136**	sheep	100	0	100	0	233	2167	0	no	Fenbendazole	licensed dose	oral	5	19
**MS142**	sheep	96	4	99	1	7717	7966	0	no	Albendazole	**2x licensed dose**	oral	10	12
**MS152**	sheep	15	85	16	84	356	333	6	no	Fenbendazole	licensed dose	oral	15	14
**MS166**	sheep	1	99	0	0	700	0	100	yes	Albendazole	licensed dose	oral	5	13
**MS167**	sheep	97	3	100	0	1200	1333	0	no	Albendazole	licensed dose	oral	5	13
**MS173**	sheep	6	94	0	100	600	17	97	yes	Albendazole	licensed dose	oral	10	11
**MS178**	sheep	5	95	100	0	433	1300	0	no	Albendazole	licensed dose	oral	5	12
**MS185**	sheep	75	25	96	4	933	367	61	no	Albendazole	licensed dose	oral	5	12
**MS188**	sheep	88	12	75	25	1233	33	97	yes	Albendazole	licensed dose	oral	5	13
**MS190**	sheep	48	52	60	40	233	67	71	no	Albendazole	licensed dose	oral	5	13
**MS2**	sheep	/	/	0	0	300	0	100	yes	Albendazole	licensed dose	oral	5	13
**MS213**	sheep	9	91	0	100	667	11	98	yes	Albendazole	licensed dose	oral	15	10
**MS22**	sheep	80	20	0	0	333	0	100	yes	Albendazole	licensed dose	oral	5	10

Flock ID	Species	Pre-treatment *Haemonchus* eggs (%)	Pre-treatment other strongyle eggs (%)	Post-treatment *Haemonchus* eggs (%)	Post-treatment other strongyle eggs (%)	Pre-treatment FEC (epg)	Post-treatment FEC (epg)	FECR (%)	Treatment success	Anthelmintic compound	Dose	Route of applica-tion	Number animals	Treatment to sampling interval (days)
**MS24**	sheep	0	100	0	0	650	0	100	yes	Albendazole	licensed dose	oral	10	15
**MS33**	sheep	35	65	0	0	1700	0	100	yes	Albendazole	licensed dose	oral	10	18
**MS35**	sheep	1	99	74	26	600	2355	0	no	Fenbendazole	licensed dose	oral	15	12
**MS37**	sheep	1	99	83	17	356	7733	0	no	Fenbendazole	licensed dose	oral	15	12
**MS4**	sheep	23	77	0	0	1000	0	100	yes	Albendazole	licensed dose	oral	5	12
**MS53**	sheep	21	79	100	0	467	33	93	no	Albendazole	licensed dose	oral	5	11
**MS62**	sheep	69	31	100	0	3389	33	99	yes	Albendazole	licensed dose	oral	15	12
**MS76**	sheep	66	34	98	2	3650	1817	50	no	Albendazole	licensed dose	oral	10	13
**MS93**	sheep	15	85	0	100	267	33	88	no	Fenbendazole	licensed dose	oral	5	17
**MS94**	sheep	38	62	10	90	233	200	14	no	Albendazole	licensed dose	oral	5	10
**MS95**	sheep	99	1	89	11	11067	3133	72	no	Albendazole	licensed dose	oral	5	14
**MS98**	sheep	93	7	88	6	3650	67	98	yes	Oxfendazole	licensed dose	oral	10	14
**S105**	sheep	50	50	100	0	1850	283	85	no	Albendazole	licensed dose	oral	10	11
**S112**	sheep	58	42	88	12	867	1633	0	no	Albendazole	licensed dose	oral	15	15
**S114**	sheep	52	48	95	5	411	689	0	no	Oxfendazole	licensed dose	oral	15	12
**S122**	sheep	75	25	0	0	1233	0	100	yes	Fenbendazole	licensed dose	oral	15	11
**S138**	sheep	16	84	71	29	967	67	93	no	Fenbendazole	licensed dose	oral	5	14
**S140**	sheep	82	18	0	0	500	0	100	yes	Albendazole	licensed dose	oral	5	13
**S143**	sheep	35	65	38	62	400	200	50	no	Oxfendazole	licensed dose	oral	5	13
**S146**	sheep	40	60	100	0	4867	267	95	yes	Albendazole	licensed dose	oral	10	11
**S150**	sheep	7	93	0	0	233	0	100	yes	Albendazole	licensed dose	oral	5	10
**S191**	sheep	29	71	0	0	1200	0	100	yes	Fenbendazole	licensed dose	oral	5	13
**S228**	sheep	89	11	92	8	1234	583	53	no	Oxfendazole	licensed dose	oral	10	11
**S241**	sheep	11	89	18	82	667	378	43	no	Fenbendazole	licensed dose	oral	15	12
**S40**	sheep	12	88	22	78	1500	633	58	no	Albendazole	licensed dose	oral	5	16

**Flock ID**	**Species**	**Pre-treatment *Haemonchus* eggs (%)**	**Pre-treatment other strongyle eggs (%)**	**Post-treatment *Haemonchus* eggs (%)**	**Post-treatment other strongyle eggs (%)**	**Pre-treatment FEC (epg)**	**Post-treatment FEC (epg)**	**FECR (%)**	**Treatment success**	**Anthelmintic compound**	**Dose**	**Route of applica-tion**	**Number animals**	**Treatment to sampling interval (days)**
**S55**	sheep	0	100	60	40	6867	333	95	yes	Albendazole	**2.5x licensed dose**	oral	5	16
**S61**	sheep	30	70	28	72	456	300	34	no	Albendazole	licensed dose	oral	15	12
**S66**	sheep	29	71	0	0	767	0	100	yes	Albendazole	licensed dose	oral	5	14
**S71**	sheep	0	100	0	0	233	0	100	yes	Albendazole	licensed dose	oral	5	14
**S90**	sheep	7	93	100	0	1034	17	98	yes	Albendazole	licensed dose	oral	10	14
**MS104**	sheep	10	90	0	0	511	0	100	yes	Closantel & Mebendazole	licensed dose	oral	15	9
**MS108**	sheep	2	98	0	0	565	0	100	yes	Closantel & Mebendazole	licensed dose	oral	15	9
**MS149**	sheep	14	86	0	100	467	233	50	no	Closantel & Mebendazole	licensed dose	oral	5	8
**MS162**	sheep	61	39	0	100	3456	11	100	yes	Closantel & Mebendazole	licensed dose	oral	15	12
**MS186**	sheep	25	75	0	100	700	33	95	yes	Closantel & Mebendazole	licensed dose	oral	5	11
**MS187**	sheep	96	4	0	0	1667	0	100	yes	Closantel & Mebendazole	licensed dose	oral	5	13
**MS91**	sheep	0	100	0	0	233	0	100	yes	Closantel & Mebendazole	licensed dose	oral	5	14
**MS96**	sheep	/	/	0	100	233	100	57	no	Closantel & Mebendazole	licensed dose	oral	5	14
**S103**	sheep	100	0	0	0	367	0	100	yes	Closantel & Mebendazole	licensed dose	oral	5	19
**S107**	sheep	28	72	0	100	2866	11	100	yes	Closantel & Mebendazole	licensed dose	oral	15	13
**S120**	sheep	100	0	0	0	1389	0	100	yes	Closantel & Mebendazole	licensed dose	oral	15	9
**S146**	sheep	70	30	0	0	633	0	100	yes	Closantel & Mebendazole	licensed dose	oral	5	10
**S149**	sheep	0	100	0	0	633	0	100	yes	Closantel & Mebendazole	licensed dose	oral	10	10
**S159**	sheep	90	10	0	100	1517	34	98	yes	Closantel & Mebendazole	licensed dose	oral	10	14
**S224**	sheep	92	8	0	100	867	11	99	yes	Closantel & Mebendazole	licensed dose	oral	15	13

Flock ID	Species	Pre-treatment *Haemonchus* eggs (%)	Pre-treatment other strongyle eggs (%)	Post-treatment *Haemonchus* eggs (%)	Post-treatment other strongyle eggs (%)	Pre-treatment FEC (epg)	Post-treatment FEC (epg)	FECR (%)	Treatment success	Anthelmintic compound	Dose	Route of applica-tion	Number animals	Treatment to sampling interval (days)
**S225**	sheep	92	8	0	0	467	0	100	yes	Closantel & Mebendazole	licensed dose	oral	5	14
**S90**	sheep	14	86	0	0	1533	0	100	yes	Closantel & Mebendazole	licensed dose	oral	5	14
**MS26 (Z)**	goat	0	100	0	100	1211	33	97	yes	Moxidectin	2x sheep dose	oral	15	14
**MZ12**	goat	12	88	0	0	400	0	100	yes	Moxidectin	2x sheep dose	oral	10	13
**MZ17**	goat	92	8	100	0	6078	1744	71	no	Moxidectin	2x sheep dose	oral	15	12
**MZ24**	goat	2	98	0	0	733	0	100	yes	Moxidectin	2x sheep dose	oral	10	14
**MZ26**	goat	64	36	0	0	1000	0	100	yes	Moxidectin	2x sheep dose	oral	15	12
**MZ39**	goat	92	8	100	0	717	50	93	no	Moxidectin	2x sheep dose	oral	10	12
**MZ43**	goat	97	3	0	0	833	0	100	yes	Moxidectin	2x sheep dose	oral	5	11
**MZ6**	goat	50	50	0	0	233	0	100	yes	Moxidectin	2x sheep dose	oral	5	10
**Z12**	goat	0	100	0	100	978	11	99	yes	Moxidectin	2x sheep dose	oral	15	14
**Z15**	goat	41	59	0	0	417	0	100	yes	Moxidectin	2x sheep dose	oral	10	12
**Z36**	goat	91	9	0	0	933	0	100	yes	Moxidectin	2x sheep dose	oral	5	11
**Z39**	goat	50	50	47	53	1122	389	65	no	Moxidectin	2x sheep dose	oral	15	13
**Z42**	goat	28	72	25	75	4322	211	95	yes	Moxidectin	2x sheep dose	oral	15	10
**Z52**	goat	79	21	0	0	2167	0	100	yes	Moxidectin	2x sheep dose	oral	5	11
**MS140MZ 28**	goat	54	46	83	17	600	67	89	no	Ivermectin	2x sheep dose	injection	10	19
**MZ35**	goat	58	42	53	47	4389	500	89	no	Ivermectin	2x sheep dose	injection	15	10
**MZ43**	goat	90	10	100	0	800	67	92	no	Eprinomectin	2x cattle dose	pour-on	5	11
**MZ21**	goat	85	15	0	100	467	50	88	no	Levamisole	1.5x sheep dose	oral	10	14
**Z48**	goat	46	54	3	97	950	117	88	no	Levamisole	2x sheep dose	oral	10	12
**Z68**	goat	43	57	0	100	989	134	86	no	Levamisole	1.5x sheep dose	oral	15	10
**MZ34**	goat	46	54	0	0	933	0	100	yes	Monepantel	2x sheep dose	oral	15	12
**Z27**	goat	81	19	100	0	622	11	98	yes	Monepantel	2x sheep dose	oral	15	11
**Z8**	goat	0	100	0	100	3922	1100	72	no	Monepantel	3x sheep dose	oral	15	14

Flock ID	Species	Pre-treatment *Haemonchus* eggs (%)	Pre-treatment other strongyle eggs (%)	Post-treatment *Haemonchus* eggs (%)	Post-treatment other strongyle eggs (%)	Pre-treatment FEC (epg)	Post-treatment FEC (epg)	FECR (%)	Treatment success	Anthelmintic compound	Dose	Route of applica-tion	Number animals	Treatment to sampling interval (days)
**MZ18**	goat	100	0	100	0	1484	450	70	no	Albendazole	3x sheep dose	oral	10	14
**MZ19**	goat	58	42	37	63	3511	2900	17	no	Fenbendazole	2x sheep dose	oral	15	14
**MZ32**	goat	1	99	3	97	1422	422	70	no	Fenbendazole	2x sheep dose	oral	15	14
**MZ41**	goat	4	96	50	50	233	300	0	no	Albendazole	2x sheep dose	oral	5	11
**MZ43**	goat	66	34	0	0	233	0	100	yes	Fenbendazole	2x sheep dose	oral	5	11
**Z15**	goat	83	17	0	0	333	0	100	yes	Albendazole	2.2x sheep dose	oral	5	11
**Z17**	goat	66	34	0	0	567	0	100	yes	Albendazole	2x sheep dose	oral	5	14
**Z36**	goat	54	46	0	0	667	0	100	yes	Albendazole	2x sheep dose	oral	5	11
**Z37**	goat	74	26	0	100	1611	22	99	yes	Albendazole	2x sheep dose	oral	15	13
**Z52**	goat	94	6	0	0	1484	0	100	yes	Fenbendazole (1 group) Albendazole (1 group)	2x sheep dose	oral	10	11
**Z54**	goat	5	95	50	50	967	67	93	no	Fenbendazole	2x sheep dose	oral	5	10
**Z61**	goat	31	69	48	52	1878	167	91	no	Fenbendazole	2x sheep dose	oral	15	11
